# Associations between body mass index and health-related physical fitness among Chinese university students: a cross-sectional study

**DOI:** 10.3389/fspor.2025.1638381

**Published:** 2025-07-15

**Authors:** Yan Li, Niujin Shi, Yimin Tao

**Affiliations:** ^1^ College of Physical Education and Health, Guangxi Normal University, Guilin, Guangxi, China; ^2^ Guilin University of Aerospace Technology, Guilin, Guangxi, China

**Keywords:** BMI, physical fitness, university students, obesity, quadratic regression

## Abstract

Obesity among university students is a growing concern with significant implications for physical and mental health. This study aims to examine the associations between body mass index (BMI) and health-related physical fitness among Chinese university students. Data were collected from 14,735 students (9,117 males and 5,618 females) aged 19–25 years. Physical fitness was assessed using the Chinese College Students' Physical Fitness Test, and BMI was calculated as weight (kg) divided by height (m)^2^. Statistical analyses included Spearman correlation and quadratic regression to explore the relationships between BMI and various physical fitness parameters. Obese students exhibited higher vital capacity but poorer performance in speed, endurance, flexibility, and strength tests compared to their normal-weight counterparts. Quadratic regression analysis revealed a non-linear relationship between BMI and physical fitness scores, with moderate BMI increases initially improving body function and physical quality scores, but further increases leading to declines. Significant sex differences were observed, with males outperforming females in strength and endurance tests, while females excelled in flexibility. Our findings underscore the importance of maintaining a healthy BMI to optimize physical fitness and overall health. Regular physical fitness assessments are essential for identifying at-risk students and implementing targeted interventions. These insights can inform public health strategies and educational programs aimed at reducing obesity prevalence and enhancing the physical well-being of university students.

## 1 Introduction

Obesity poses significant risks to both physical (1) and mental health (2), including cardiovascular diseases (3), metabolic disorders (4), cancer (5), and psychological issues such as anxiety and depression (6). Additionally, obesity can impair cognitive functions and learning abilities (7, 8), highlighting the importance of regular health assessments in this demographic to address immediate and long-term health risks.

Assessing physical fitness and body composition is crucial for identifying health risks early and implementing preventive measures (9–11). In particular, body mass index (BMI) is a widely recognized metric that provides a straightforward means of classifying weight categories and assessing associated health risks (12–14). Zhong et al. analyzed data from over 89,000 participants to explore the impact of metabolomic BMI phenotypes on health outcomes, finding that individuals with obesity-related metabolites had a significantly higher risk of mortality and morbidity compared to those with normal health metabolites (15). In a large prospective cohort study involving 121,799 middle-aged adults from the UK Biobank, Heianza et al. found that individuals with a higher genetic risk for obesity, as measured by a BMI-associated genetic risk score, were more likely to develop cardiovascular disease (CVD) (16).This underscores the utility of BMI as a vital tool in health evaluations.

Monitoring the health status of students is a priority in physical education and public health (17). Regular assessments inform targeted health promotion strategies and support initiatives aimed at reducing obesity prevalence (18, 19). By prioritizing student health, educational institutions can significantly contribute to developing a healthier and more active generation (20–22).

This study investigates the relationship between BMI and health-related physical fitness among Chinese university students. By examining these associations, the research aims to inform public health interventions and improve student health outcomes. The findings will provide valuable insights into promoting effective health strategies and enhancing the overall well-being of university students.

## 2 Materials and methods

### 2.1 Participants

**Figure 1:**
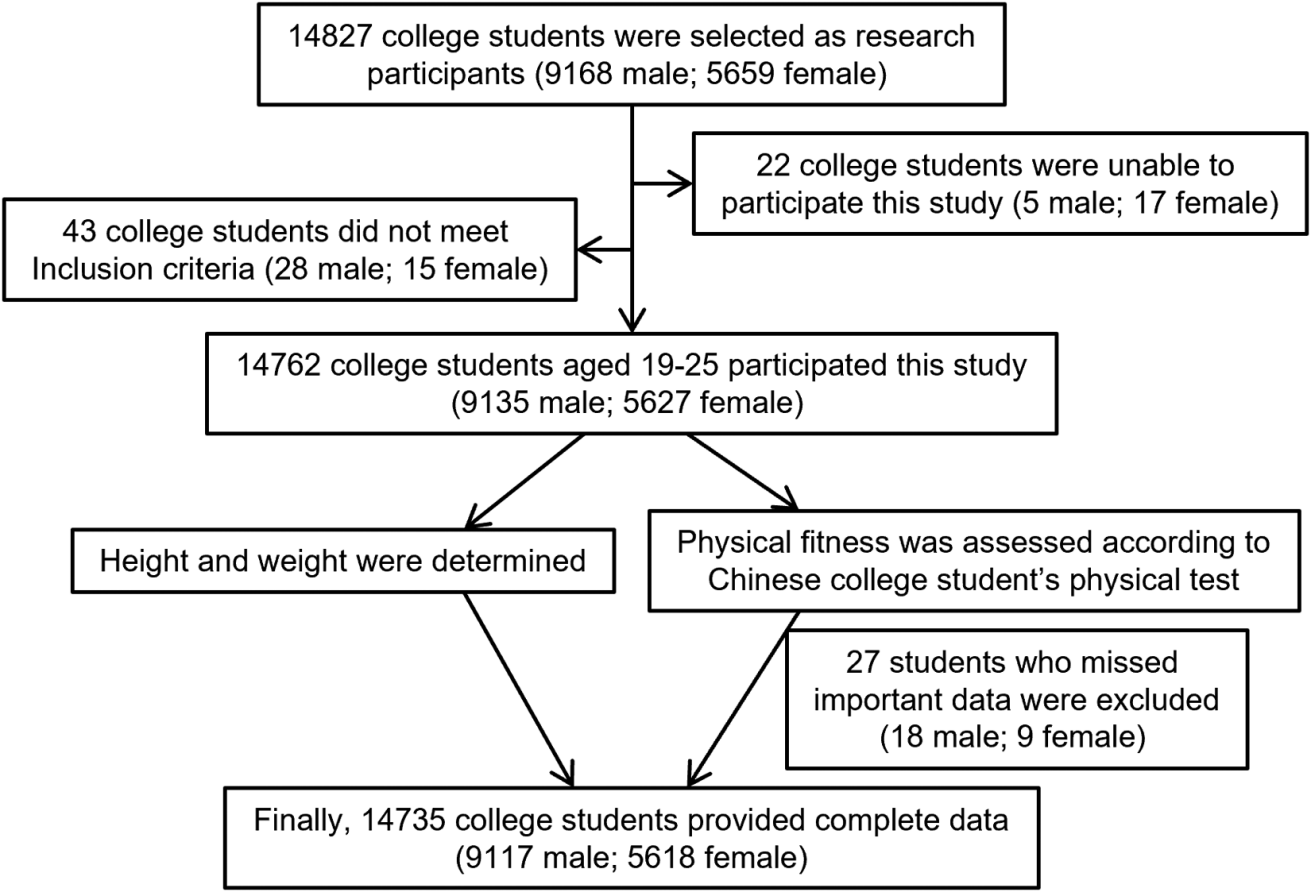
Flowchart of the study enrollment.

This cross-sectional study was conducted as a census of all enrolled undergraduates during the 2022 academic year at Guilin University of Aerospace Technology. A total of 14,827 students were initially selected to participate in the physical fitness assessments. Among all participants, 22 were unable to complete the assessments due to emergency situations. Additionally, 43 participants with cardiovascular, respiratory, or metabolic diseases were excluded from the study. As a result, 14,762 students took part in the assessments. During the testing process, data from 27 participants were partially missing, leading to their exclusion from the final analysis. Consequently, complete data were obtained from 14,735 participants (9,117 males, 5,618 females). All participants provided informed consent. A flowchart of the study process is presented in Figure 1. The study protocol adhered to the principles of the Declaration of Helsinki and was approved by the Ethics Committee of Guangxi Normal University (Approval Number: 20231226001).

### 2.2 BMI measurement

BMI was calculated for each participant using the standard formula: BMI = weight (kg)/height (m)^2^. Height was measured to the nearest 0.1 cm using a stadiometer with participants standing barefoot, and weight was measured to the nearest 0.1 kg using a calibrated digital scale with participants wearing light clothing (23).

### 2.3 Physical fitness test

The physical fitness of participants was assessed using a standardized battery of tests. Each test was conducted according to established protocols, and participants were given adequate time to rest between tests to prevent fatigue.

#### 2.3.1 Vital capacity

Vital capacity was measured using a portable spirometer. Participants were instructed to take a deep breath and exhale as forcefully as possible into the spirometer. The highest value from three trials was recorded as the vital capacity.

#### 2.3.2 50-m sprint

The 50-meter sprint test measured participants’ speed and acceleration. Participants were instructed to run the distance as quickly as possible from a standing start. The time was recorded to the nearest 0.01 s using electronic timing gates.

#### 2.3.3 Sit and reach

The sit and reach test was used to assess lower back and hamstring flexibility. Participants sat on the floor with legs fully extended and reached forward along a measuring line as far as possible. The distance reached was recorded to the nearest 0.1 cm.

#### 2.3.4 Standing long jump

The standing long jump test evaluated lower body explosive strength. Participants were instructed to jump as far forward as possible from a standing position, using both feet to take off. The distance from the starting line to the nearest point of contact on landing was measured to the nearest 0.1 cm.

#### 2.3.5 Pull-up/bent-leg sit-up

For male participants, upper body strength was assessed using the pull-up test. Participants were required to perform as many pull-ups as possible with proper form. For female participants, core strength was evaluated using the bent-leg sit-up test. Participants were instructed to perform as many sit-ups as possible within one minute, with knees bent at a 90-degree angle.

#### 2.3.6 1,000/800-m run

Cardiorespiratory endurance was assessed using a timed run. Male participants completed a 1,000-meter run, while female participants completed an 800-meter run. The time to complete the run was recorded to the nearest 0.1 s.

### 2.4 Physical fitness score

According to the “National Student Physical Fitness Standards,” BMI is categorized into four levels. For males, the BMI classifications are: underweight (≤17.8 kg/m²), normal weight (17.9–23.9 kg/m²), overweight (24.0–27.9 kg/m²), and obese (≥28.0 kg/m²). For females, the classifications are: underweight (<17.1 kg/m²), normal weight (17.2–23.9 kg/m²), overweight (24.0–27.9 kg/m²), and obese (>28.0 kg/m²). Students with a normal BMI receive a score of 100, those classified as underweight or overweight receive 80, and those classified as obese receive 60. The detailed BMI scoring criteria are provided in Supplementary Table S1 (23).

The physical fitness score is composed of three components: body composition (BMI, contributing 15 points), physiological function (vital capacity, contributing 15 points), and physical fitness (including sit and reach, 10 points; standing long jump, 10 points; pull-up or bent-leg sit-up, 10 points; 50-m sprint, 20 points; and 800-m or 1,000-m run, 20 points). The total physical fitness score is calculated by summing the weighted scores of each component. The formula is: Total Score = Body Composition Score + Physiological Function Score + Physical Fitness Score = (BMI Score × 15%) + (Vital Capacity Score × 15%) + [(Sit and Reach Score × 10%) + (Standing Long Jump Score × 10%) + (Pull-Up/Bent-Leg Sit-Up Score × 10%) + (50-m Sprint Score × 20%) + (800/1,000-m Run Score × 20%)] (Supplementary Table S2) (23).

### 2.5 Statistical analysis

A two-way analysis of variance (ANOVA) was conducted to assess differences in physical fitness indicators based on gender and BMI categories. An independent-sample *t*-test was conducted to compare the mean difference among gender groups. Spearman correlation analysis was used to evaluate the linear relationship between BMI and physical fitness scores. Quadratic regression analysis was employed to assess the nonlinear relationship between BMI and physical fitness scores. *p* < 0.05 was considered as statistically significant. All data were analyzed using the GraphPad Prism (GraphPad Prism 8.02, San Diego, CA, USA).

## 3 Results

### 3.1 Participants’ characteristics

The study included a total of 14,735 participants, with 9,117 males and 5,618 females. Participants were categorized based on their BMI into four groups: underweight, normal weight, overweight, and obese. The distribution of age, body shape data, and physical fitness test results across these BMI categories is summarized in Table 1.

**Table 1:** Descriptive characteristics of the participants.

Variables	All (*n* = 14,735)	Male (*n* = 9,117)				Female (*n* = 5,618)			
		Underweight (*n* = 1,019)	Normal weight (*n* = 6,068)	Overweight (*n* = 1,405)	Obese (*n* = 625)	Underweight (*n* = 642)	Normal weight (*n* = 4,401)	Overweight (*n* = 429)	Obese (*n* = 146)
Age (years) [*n* (%)]									
≤ 20	1,891 (12.83)	159 (15.60)	727 (11.98)	215 (15.30)	103 (16.48)	61 (9.50)	541 (12.29)	58 (13.52)	27 (18.49)
21	3,718 (25.23)	272 (26.69)	1,597 (26.32)	339 (24.13)	166 (26.56)	146 (22.74)	1,050 (23.86)	110 (25.64)	38 (26.03)
22	3,975 (26.98)	268 (26.30)	1,703 (28.07)	398 (28.33)	186 (29.76)	167 (26.01)	1,130 (25.68)	93 (21.68)	30 (20.55)
23	3,062 (20.78)	200 (19.63)	1,260 (20.76)	261 (18.58)	110 (17.60)	153 (23.83)	949 (21.56)	96 (22.38)	33 (22.60)
≥ 24	2,089 (14.18)	120 (11.78)	781 (12.87)	192 (13.67)	60 (9.60)	115 (17.91)	731 (16.61)	72 (16.78)	18 (12.33)
Body shape data [mean (SD)]									
Height (cm)	166.6 (8.8)	171.1 (6.1)	170.8 (7.4)	171.6 (6.1)	172.3 (6.4)	159.7 (5.5)	159.2 (6.0)	158.6 (5.9)	161.3 (5.8)
Weight (kg)	58.87 (12.65)	49.60 (4.31)	60.19 (6.95)	75.29 (6.52)	92.08 (10.73)	41.68 (3.24)	50.53 (5.72)	64.18 (5.82)	79.78 (8.48)
BMI (kg/m^2^)	21.10 (3.57)	16.92 (0.79)	20.56 (1.64)	25.52 (1.13)	30.98 (2.56)	16.33 (0.68)	19.90 (1.70)	25.47 (1.12)	30.64 (2.38)
Physical fitness test data [mean (SD)]									
Vital capacity (ml)	3,276 (832)	3,380 (544)	3,719 (630)	3,974 (636)	4,066 (708)	2,306 (363)	2,519 (435)	2,688 (468)	2,870 (504)
50-m sprint (s)	8.60 (1.10)	7.91 (0.58)	7.81 (0.60)	8.06 (0.59)	8.46 (0.72)	9.66 (0.66)	9.69 (0.72)	9.91 (0.70)	10.20 (1.06)
Sit and reach (cm)	15.81 (6.86)	12.10 (6.67)	14.80 (6.91)	14.86 (6.68)	13.54 (6.94)	16.57 (6.29)	18.15 (6.29)	18.02 (6.06)	16.44 (6.21)
Standing long jump (cm)	198.3 (33.7)	222.0 (19.6)	222.8 (21.3)	211.5 (20.7)	197.3 (22.9)	167.0 (16.7)	165.0 (17.4)	157.8 (17.7)	151.3 (17.9)
Pull up (times)	–	8.53 (4.50)	7.76 (5.31)	3.79 (4.23)	1.14 (1.95)	–	–	–	–
Bent-leg sit-up (times)	–	–	–	–	–	31.98 (7.00)	32.63 (7.57)	31.50 (7.00)	30.48 (7.37)
1,000-m run (s),	–	262.3 (25.1)	255.6 (31.0)	271.5 (34.9)	314.0 (41.7)	–	–	–	–
800-m run (s)	–	–	–	–	–	257.7 (25.5)	257.9 (27.4)	264.8 (28.9)	289.9 (39.1)
Physical fitness score [mean (SD)]									
Body shape score	13.97 (1.72)	13.79 (1.84)				14.27 (1.46)			
Body function score	9.94 (2.61)	8.65 (2.70)	9.93 (2.43)	10.80 (2.04)	11.02 (2.42)	8.83 (3.00)	9.87 (2.77)	10.75 (2.18)	11.41 (2.33)
Physical quality score	43.73 (7.78)	43.95 (6.06)	44.45 (6.95)	38.94 (6.82)	30.64 (8.54)	46.10 (6.29)	46.06 (6.78)	42.95 (7.57)	35.89 (11.26)
Total score of physical fitness	67.65 (8.80)	64.60 (6.67)	69.38 (7.36)	61.75 (7.22)	50.66 (9.36)	66.93 (6.92)	70.92 (7.34)	65.69 (7.93)	56.30 (11.76)

BMI, body mass index; SD, standard deviation; cm, centimeter; kg, kilogram; m, meter; ml, milliliter; s, second.

Most participants were 21–22 years old, accounting for approximately 52% of the total sample. Vital capacity, sprint time, flexibility, jump distance, and strength were measured for all participants. Obese participants generally demonstrated higher vital capacity values, with obese males averaging 4,066 ml and obese females 2,870 ml. Conversely, underweight participants showed lower scores in these tests. Notably, sprint times and endurance measures, such as the 1,000-m and 800-m runs, were slower in overweight and obese groups compared to normal weight participants. The overall physical fitness scores showed a decline with increasing BMI. Normal weight participants had the highest average total physical fitness score (69.38 for males and 70.92 for females), whereas obese participants had the lowest (61.75 for males and 56.30 for females).

**Figure 2:**
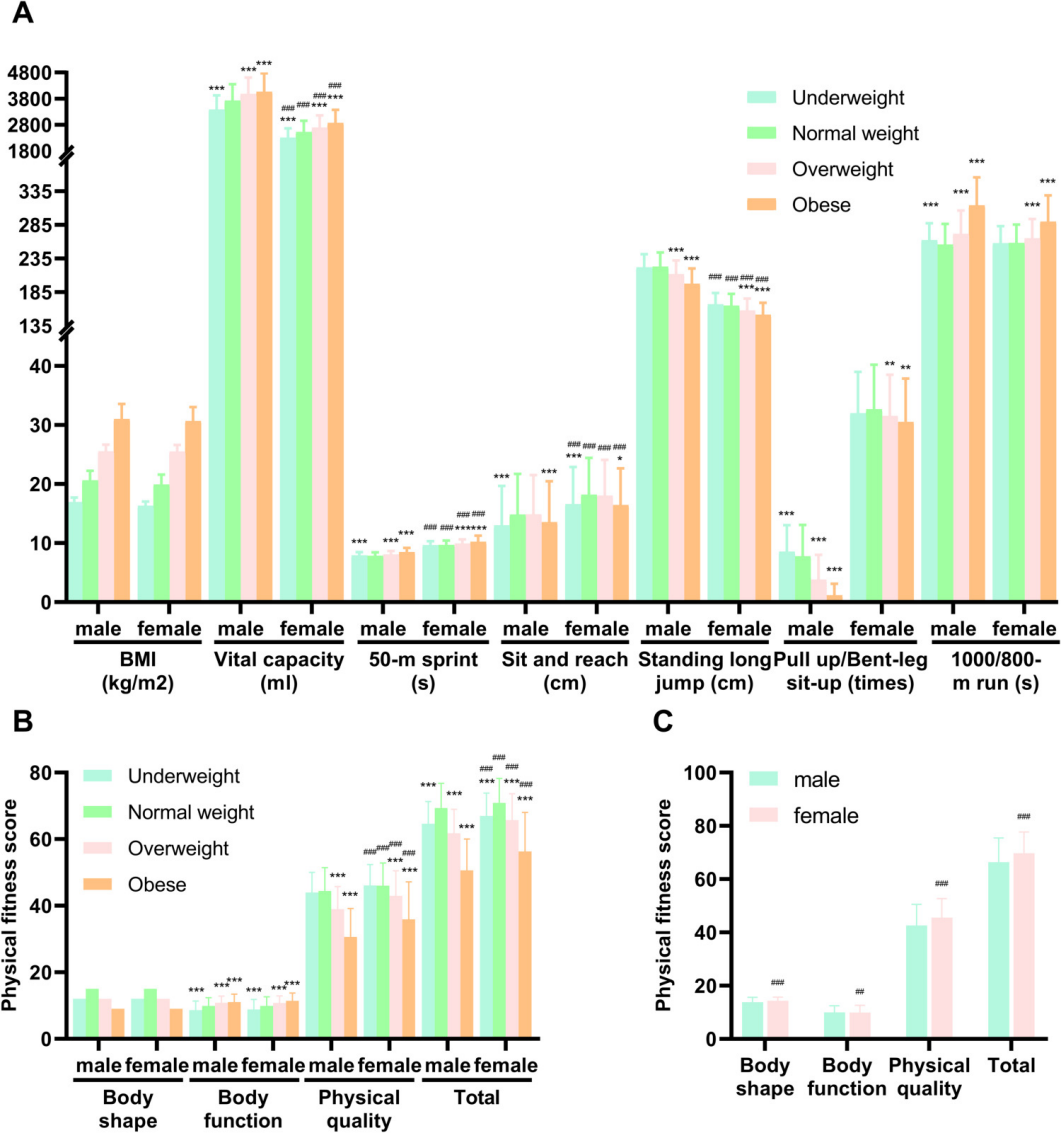
Comparison of physical fitness test **(A)** and physical fitness score **(B,C)** in different BMI status and sex. Data of **(A,B)** were analyzed using two-way ANOVA, and data of **(C)** were analyzed using unpaired *t*-test. BMI = weight (kg)/height (m)^2^; total (total physical fitness) = body shape score + body function score + physical quality score = BMI score × 15% + vital capacity score × 15% + (50-m sprint score × 20% + sit and reach score × 10% + standing long jump score × 10% + pull-up/bent-leg sit-up score × 10% + 800/1,000-m run score × 20%). All values are mean ± SD. Normal weight as reference * *p* < 0.05; ** *p* < 0.01; *** *p* < 0.001. Male as reference ^#^ *p* < 0.05; ^##^ *p* < 0.01; ^###^ *p* < 0.001. BMI, body mass index; SD, standard deviation; cm, centimeter; kg, kilogram; m, meter; ml, milliliter; s, second.

Obese individuals exhibited higher vital capacity compared to normal weight (Figure 2A). However, they performed worse in speed and endurance-related tests, such as the 50-m sprint and 800/1,000-m run. In terms of flexibility and muscle strength, as assessed by the sit-and-reach and pull-up/bent-leg sit-up tests, obese participants generally scored lower than their normal-weight peers. When comparing physical fitness scores, normal-weight individuals outperformed those in the other BMI categories, particularly in body function and physical quality (Figure 2B). Sex differences were also evident, with males generally outperforming females in tests requiring strength and endurance, such as the pull-up and 1,000-m run. However, females showed superior performance in flexibility tests like the sit-and-reach (Figure 2C).

### 3.2 Correlation analysis between BMI and physical fitness

**Figure 3:**
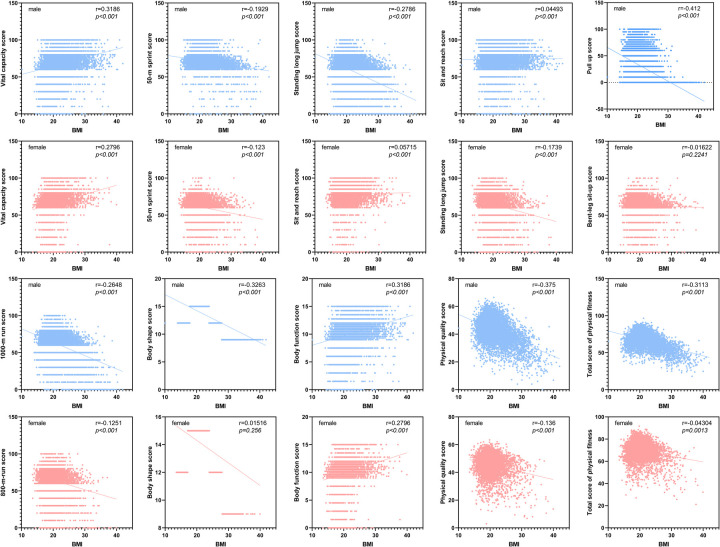
Spearman correlation analysis between BMI and various physical fitness score. BMI = weight (kg)/height (m)^2^; total (total physical fitness) = body shape score + body function score + physical quality score = BMI score × 15% + vital capacity score × 15% + (50-m sprint score × 20% + sit and reach score × 10% + standing long jump score × 10% + pull-up/bent-leg sit-up score × 10% + 800/1,000-m run score × 20%). BMI, body mass index.

Figure 3 illustrates the Spearman correlation analysis between BMI and various physical fitness scores. The Spearman correlation coefficients reveal significant relationships between BMI and the different components of physical fitness. As BMI increases, there is a notable inverse correlation with overall physical fitness scores. Specifically, higher BMI values are associated with lower scores in the 50-m sprint, sit and reach, standing long jump, and the 800/1,000-m run, indicating diminished speed, flexibility, explosive strength, and endurance. Conversely, the correlation between BMI and vital capacity is positive, suggesting that higher BMI is associated with greater vital capacity.

### 3.3 Regression analysis between BMI and physical fitness

**Figure 4:**
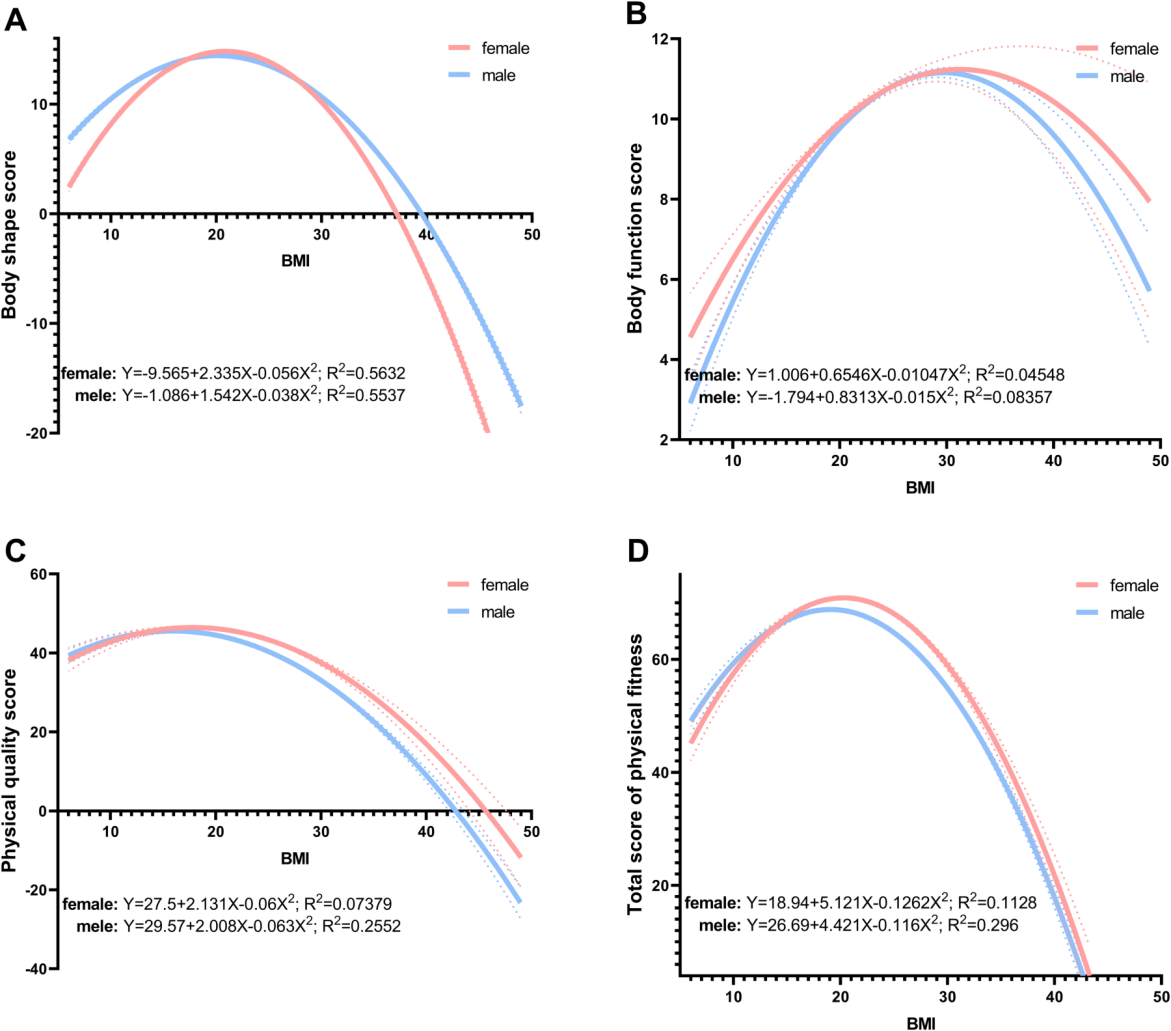
Quadratic regression analysis of BMI and body shape score **(A)**, body function score **(B)**, physical quality score **(C)**, total score of physical fitness **(D)** dashed lines represent the 95% CI. BMI = weight (kg)/height (m)2; total (total physical fitness) = body shape score + body function score + physical quality score = BMI score × 15% +vital capacity score × 15% + (50-m sprint score × 20% + sit and reach score × 10% + standing long jump score × 10% + pull-up/bent-leg sit-up score × 10% + 800/1,000-m run score × 20%). CI, confidence interval; BMI, body mass index.

Following the correlation analysis, we conducted a regression analysis to further investigate the relationship between BMI and physical fitness. Figure 4 presents the quadratic regression models for body shape score (A), body function score (B), physical quality score (C), and the total score of physical fitness (D). For body shape score, the female model (R² = 0.5632) yields a turning point at approximately BMI = 20.58 kg/m², whereas the male model (R² = 0.5537) peaks near BMI = 20.28 kg/m². This suggests that moderate increases in BMI up to these values are associated with improved body shape metrics, but scores decline beyond them. In the case of body function score, the female model (R² = 0.04548) has a lower R-squared value compared to the male model (R² = 0.08357), the models indicate optimal BMI values around 31.26 kg/m² for females and 27.71 kg/m² for males. Interventions aiming to enhance functional capacity should therefore prioritize individuals whose BMI deviates substantially from these peaks. The physical quality score regression models show a similar pattern, with the female model (R² = 0.07379) again fitting the data better than the male model (R² = 0.2552), the turning points occur at BMI = 17.76 kg/m^2^ (female) and 15.9 kg/m^2^ (male), highlighting a similar window for maximizing overall physical quality. The total score of physical fitness, calculated using a weighted formula, also demonstrates a non-linear relationship with BMI. The female model (R² = 0.1128) and male model (R² = 0.296) peaks at BMI = 20.28 kg/m^2^ for females and BMI = 19.06 kg/m^2^ for males. This analysis highlights the complexity of the BMI-fitness relationship and the potential for optimization at specific BMI levels. Additionally, Supplementary Figure S1 provides a comprehensive quadratic regression analysis for other physical fitness components, including vital capacity, 50-m sprint, sit and reach, standing long jump, pull-up/bent-leg sit-up, and the 1,000/800-m run.

## 4 Discussion

Our study investigated the associations between BMI and health-related physical fitness among Chinese university students, involving a substantial sample size of 14,735 participants. The primary findings reveal a clear relationship between higher BMI and poorer performance in various physical fitness tests, with significant sex differences observed.

Our results indicate that obese students tend to have higher vital capacity yet perform worse in speed, endurance, flexibility, and strength tests compared to their normal-weight counterparts. This aligns with findings by Ortega et al., who conducted a study on European adolescents and found that higher BMI was significantly associated with lower cardiorespiratory fitness, indicating the adverse impact of obesity on physical health (24). The higher vital capacity observed in obese individuals could be attributed to increased respiratory muscle strength due to greater body mass. First, increased adipose tissue on the chest wall and abdomen elevates the work of breathing, which can chronically strengthen the diaphragm and intercostal muscles, thereby augmenting maximal inspiratory and expiratory efforts. Second, greater body mass is often accompanied by enlarged lung parenchymal volume and alveolar surface area, potentially raising total lung capacity and vital capacity measurements, even though airway resistance may be higher. Third, obesity related increases in circulating blood volume and pulmonary capillary pressures can lead to subtle pulmonary vascular remodeling, which may expand lung blood volume and contribute to larger measured lung volumes. This relationship has been demonstrated in studies like the one by Harik-Khan et al., which analyzed respiratory function across different BMI categories in a cohort of 8,000 adults and found that increased body mass is associated with higher lung volumes but potentially reduced airflow (25). Understanding these specific associations is crucial for designing targeted health interventions aimed at improving physical fitness and reducing obesity-related health risks, thereby contributing to public health efforts to foster healthier student populations (17, 18).

Interestingly, our quadratic regression analysis showed a non-linear relationship between BMI and physical fitness scores. Moderate increases in BMI initially correlated with improvements in body function and physical quality scores, but further increases led to declines (26). This is consistent with findings from Pribis et al., who analyzed physical fitness and BMI in 1,256 U.S. university students. Their study demonstrated that overweight students performed better in strength-related tests but significantly worse in agility and endurance tests compared to their normal-weight counterparts, with obese students showing the poorest overall performance (27). The initial improvement in physical quality scores with slight increases in BMI could be due to increased muscle mass, which enhances strength and endurance (28). However, excessive BMI likely introduces metabolic and cardiovascular burdens, leading to reduced overall fitness (29). These insights emphasize the need for balanced nutritional and physical activity programs in universities to help students maintain an optimal BMI, which is essential for long-term health and well-being (30, 31).

Sex differences were evident, with males generally outperforming females in strength and endurance tests, while females excelled in flexibility. This is consistent with the findings by Ben Mansour G, who analyzed 136 university students and found that males generally outperformed females in strength and power tests due to their greater muscle mass, while females showed better flexibility, likely attributable to differences in joint structure and connective tissue composition (32). Beyond differences in muscle mass and connective-tissue structure, endogenous hormone profiles—particularly higher circulating testosterone in males—contribute to greater muscle hypertrophy and anaerobic capacity, while estrogen in females may enhance ligament laxity and flexibility. Moreover, gender-based socialization influences exposure to physical activities from an early age: boys are often encouraged toward strength and competitive sports, whereas girls may receive more opportunities in dance or gymnastics, reinforcing divergent motor skill development. Recognizing these sex-specific differences is vital for developing tailored fitness programs that address the unique needs of male and female students, thereby enhancing overall fitness and promoting gender-specific health strategies in educational institutions (31, 33). Building on our findings—particularly the identified optimal BMI windows and sex specific performance profiles—educational institutions can implement tiered, evidence-based interventions. For instance, a BMI-stratified campus fitness program might assign students to tailored exercise modules: (1) underweight (<18.5 kg/m²): Resistance-focused circuits to promote lean mass accrual, combined with high-protein nutrition workshops; (2) normal weight (18.5–24.9 kg/m²): Mixed aerobic-resistance routines to maintain fitness, supplemented by flexibility and balance clinics; (3) overweight/Obese (≥25 kg/m²): Progressive interval training (e.g., HIIT) plus dietary counseling sessions. To enhance engagement and monitoring, technology-based approaches can be integrated: (1) activity-tracking apps (e.g., Fitbit, smartphone pedometer apps) enable real-time feedback on step counts, heart rate zones, and workout duration, with automated reminders to reduce sedentariness; (2) gamification elements, such as team challenges or point systems, foster peer support and healthy competition; (3) wearable devices can feed data into a centralized dashboard, allowing health educators to flag individuals deviating from target activity or BMI ranges and deliver just-in-time interventions (e.g., push notifications to attend strength training sessions).

While our cross-sectional design and reliance on self-reported measures limit the ability to draw causal inferences, they also open the possibility of selection bias and residual confounding. For example, participants with higher health consciousness may have been more likely to volunteer, and unmeasured lifestyle factors—such as habitual physical activity patterns, dietary intake, or socioeconomic status—could influence both BMI and fitness outcomes. Consequently, any observed associations should be interpreted as correlational rather than causal. Moreover, because our participants were recruited from a single university, regional and institutional factors may limit the generalizability of these findings to the broader population of Chinese college students. Building on our cross-sectional findings, future research should prioritize longitudinal tracking: regularly measure BMI and fitness (e.g., quarterly tests) to see how changes in body composition affect capacities like vital capacity and sprint times; targeted interventions: conduct randomized trials in BMI defined groups (normal vs. overweight/obese), comparing tailored exercise or diet programs on outcomes such as muscle strength and endurance; psychological links: add surveys on body image and well-being alongside physical tests to explore how BMI and fitness shifts interact with student mental health.In conclusion, our study underscores the significant associations between BMI and health-related physical fitness among Chinese university students. These findings highlight the importance of maintaining a healthy BMI to optimize physical fitness and overall health. The study provides valuable insights that can inform public health strategies and educational programs aimed at reducing obesity prevalence and enhancing the physical well-being of university students. By integrating regular fitness assessments into the educational system, institutions can play a crucial role in fostering a healthier and more active generation, ultimately leading to improved public health outcomes.

## Data Availability

The original contributions presented in the study are included in the article/Supplementary Material, further inquiries can be directed to the corresponding author.
