# Supplementary material for: Associations between body mass index and health-related physical fitness among Chinese university students: a cross-sectional study
Source: Front Sports Act Living. 2025 Jul 15;7:1638381. doi: 10.3389/fspor.2025.1638381 (PMC12303885; doi:10.3389/fspor.2025.1638381)
Supplement: Supplementary file 1 [file Datasheet1.docx]

Supplementary Material

# Supplementary Tables

| **Table S1. Scoring standards of BMI** | | | |
| --- | --- | --- | --- |
| Grade | Score | BMI (kg/m^2^) | |
|  |  | Male | Female |
| Normal weight | 100 | 17.9 ~ 23.9 | 17.2 ~ 23.9 |
| Underweight | 80 | ≤ 17.8 | ≤ 17.1 |
| Overweight |  | 24.0 ~ 27.9 | 24.0 ~ 27.9 |
| Obese | 60 | ≥ 28.0 | ≥ 28.0 |

BMI body mass index; BMI = weight (kg)/height (m)^2^

| **Table S2. The national standards for students' physical health** | | | | | | | | | | | | | |
| --- | --- | --- | --- | --- | --- | --- | --- | --- | --- | --- | --- | --- | --- |
| Grade | Score | Vital capacity (ml) | | 50-m sprint (s) | | Sit and reach (cm) | | Standing long jump (cm) | | Pull up / Bent-leg sit-up (times) | | 1000 / 800-m run (s) | |
|  |  | Male | Female | Male | Female | Male | Female | Male | Female | Male | Female | Male | Female |
| Excellent | 100 | 5040 | 3400 | 6.7 | 7.5 | 24.9 | 25.8 | 273 | 207 | 19 | 56 | 197 | 198 |
|  | 95 | 4920 | 3350 | 6.8 | 7.6 | 23.1 | 24.0 | 268 | 201 | 18 | 54 | 202 | 204 |
|  | 90 | 4800 | 3300 | 6.9 | 7.7 | 21.3 | 22.2 | 263 | 195 | 17 | 52 | 207 | 210 |
| Good | 85 | 4550 | 3150 | 7.0 | 8.0 | 19.5 | 20.6 | 256 | 188 | 16 | 49 | 214 | 217 |
|  | 80 | 4300 | 3000 | 7.1 | 8.3 | 17.7 | 19.0 | 248 | 181 | 15 | 46 | 222 | 224 |
| Pass | 78 | 4180 | 2900 | 7.3 | 8.5 | 16.3 | 17.7 | 244 | 178 |  | 44 | 227 | 229 |
|  | 76 | 4060 | 2800 | 7.5 | 8.7 | 14.9 | 16.4 | 240 | 175 | 14 | 42 | 232 | 234 |
|  | 74 | 3940 | 2700 | 7.7 | 8.9 | 13.5 | 15.1 | 236 | 172 |  | 40 | 237 | 239 |
|  | 72 | 3820 | 2600 | 7.9 | 9.1 | 12.1 | 13.8 | 232 | 169 | 13 | 38 | 242 | 244 |
|  | 70 | 3700 | 2500 | 8.1 | 9.3 | 10.7 | 12.5 | 228 | 166 |  | 36 | 247 | 249 |
|  | 68 | 3580 | 2400 | 8.3 | 9.5 | 9.3 | 11.2 | 224 | 163 | 12 | 34 | 252 | 254 |
|  | 66 | 3460 | 2300 | 8.5 | 9.7 | 7.9 | 9.9 | 220 | 160 |  | 32 | 257 | 259 |
|  | 64 | 3340 | 2200 | 8.7 | 9.9 | 6.5 | 8.6 | 216 | 157 | 11 | 30 | 262 | 264 |
|  | 62 | 3220 | 2100 | 8.9 | 10.1 | 5.1 | 7.3 | 212 | 154 |  | 28 | 267 | 269 |
|  | 60 | 3100 | 2000 | 9.1 | 10.3 | 3.7 | 6.0 | 208 | 151 | 10 | 26 | 272 | 274 |
| Fail | 50 | 2940 | 1960 | 9.3 | 10.5 | 2.7 | 5.2 | 203 | 146 | 9 | 24 | 292 | 284 |
|  | 40 | 2780 | 1920 | 9.5 | 10.7 | 1.7 | 4.4 | 198 | 141 | 8 | 22 | 312 | 294 |
|  | 30 | 2620 | 1880 | 9.7 | 10.9 | 0.7 | 3.6 | 193 | 136 | 7 | 20 | 332 | 304 |
|  | 20 | 2460 | 1840 | 9.9 | 11.1 | -0.3 | 2.8 | 188 | 131 | 6 | 18 | 352 | 314 |
|  | 10 | 2300 | 1800 | 10.1 | 11.3 | -1.3 | 2.0 | 183 | 126 | 5 | 16 | 372 | 324 |

For male students, the pull-up and 1000-meter run are administered, while female students are assessed with the bent-leg sit-up and 800-meter run

# Supplementary Figures


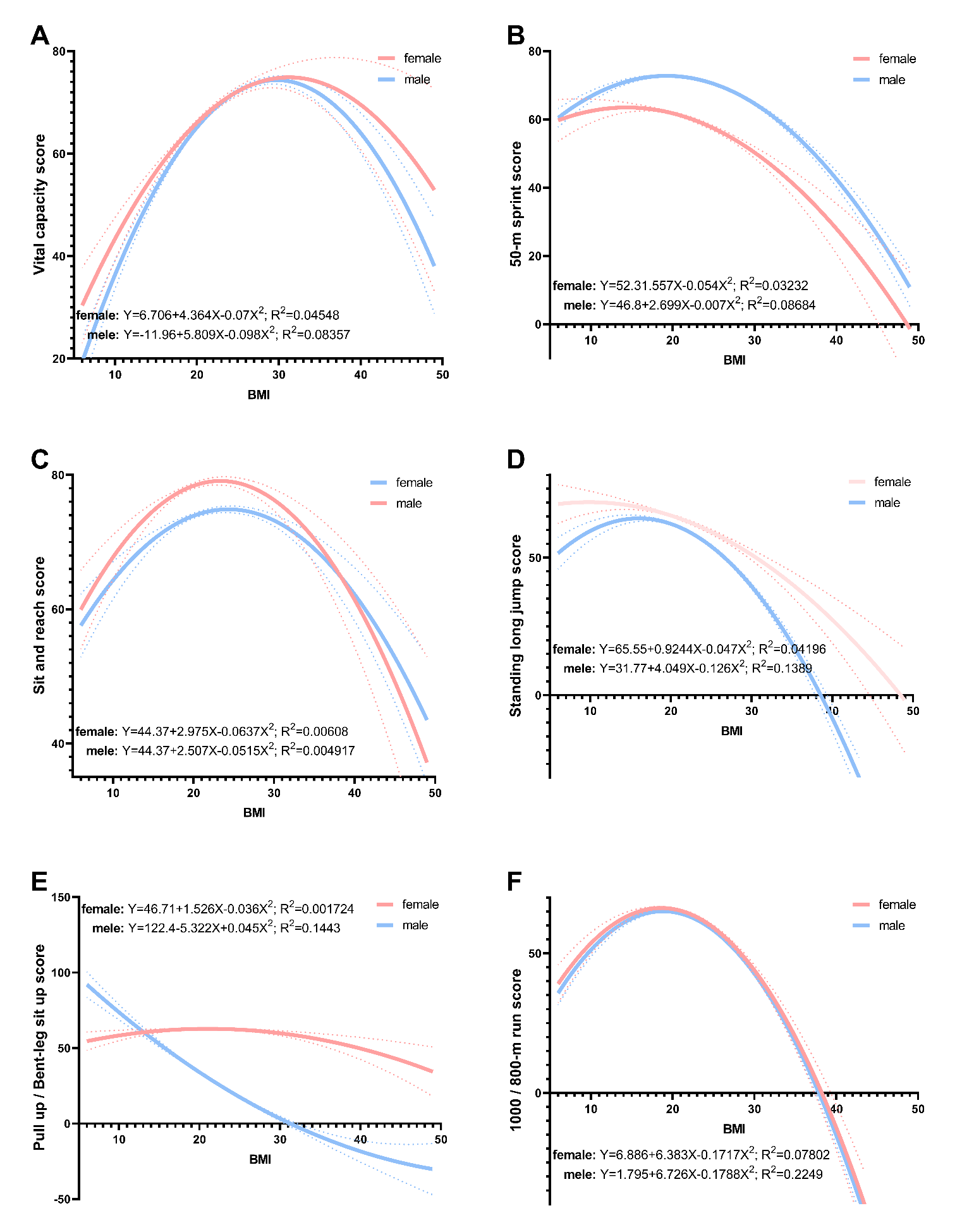


**Figure S1.** Quadratic Regression Analysis of BMI and vital capacity (A), 50-m sprint (B), sit and reach (C), standing long jump (D), Pull up / Bent-leg sit-up (E), 1000 / 800-m run (F). Dashed lines represent the 95% CI. BMI = weight (kg)/height (m)^2^. CI: confidence interval; BMI: body mass index.
